# Neurocognitive outcomes of HIV-associated tuberculous meningitis

**DOI:** 10.12688/wellcomeopenres.16967.1

**Published:** 2021-08-12

**Authors:** Carson M Quinn, John Kasibante, Alice Namudde, Ananta S Bangdiwala, Mable Kabahubya, Noeline Nakasujja, Sarah Lofgren, Alison Elliott, David R Boulware, David B Meya, Fiona V Cresswell

**Affiliations:** 1 School of Medicine, University of California San Francisco Medical Center, San Francsisco, CA, USA; 2 Infectious Diseases Institute, College of Health Sciences, Makerere University, Kampala, Uganda; 3 Department of Medicine, Brigham and Women's Hospital, Boston, MA, USA; 4 Department of Biostatistics, University of Minnesota, Minneapolis, MN, USA; 5 Department of Medicine, School of Medicine, College of Health Sciences, Makerere University, Kampala, Uganda; 6 Department of Psychiatry, School of Medicine, College of Health Sciences, Makerere University, Kampala, Uganda; 7 Division of Infectious Diseases and International Medicine, University of Minnesota, Minneapolis, Minnesota, 55455, USA; 8 Clinical Research Department, London School of Hygiene & Tropical Medicine, London, UK; 9 Medical Research Council/Uganda Virus Research-Institute Uganda Research Unit on AIDS, Entebbe, Uganda; 10 Division of Global Health and Infection, Brighton and Sussex Medical School, University of Sussex, UK

**Keywords:** Tuberculous Meningitis, HIV, neurocognitive, functional, psychiatric, depression

## Abstract

**Background: **The toll of tuberculous meningitis (TBM) in both mortality and disability is considerable, but advancements in rehabilitation have the potential to improve the functional abilities and the quality of survivors’ lives. However, the typical phenotype of neurocognitive impairment in TBM survivors remains unstudied in HIV-predominant populations in sub-Saharan Africa.

**Methods:** We tested 36 survivors of TBM in Uganda with a comprehensive battery of neurocognitive assessments at 8 and 24 weeks after diagnosis, and compared results to a representative cohort of HIV-uninfected Ugandans.

**Results:** While participants had a broad range of impairments at eight weeks, there was marked improvement by 24 weeks, when a phenotype of impairment including deficits in motor functioning, verbal learning and memory, processing speed, and executive function emerged. These deficits were present despite good clinician-rated functional status. The majority (23/27, 85%) had evidence of moderate to severe depression at week 8, and at week 24 (18/24, 75%).

**Conclusion:** These findings highlight the need for more comprehensive neurocognitive assessment in the survivors of TBM, and further investment in and study of rehabilitation, including management of depression, to improve long-term outcomes in this population.

## Introduction

Tuberculous meningitis (TBM) continues to incur unacceptably high mortality, especially in people living with HIV, in whom it can exceed 50% ^1, 2^. The persistence of neurologic sequelae in those who survive has been long-recognized, and can include major neurologic deficits such as hemiplegia and blindness, as well as more subtle cognitive changes such as memory or psychiatric problems ^3, 4^. The various neurologic sequelae have been reported to affect a third to a half of survivors in some series ^1, 2^. These long-term neurological complications are attributed to hydrocephalus ^5^, decreased grey matter volume ^6^, and stroke, which may occur in as many as 57% of patients ^7, 8^.

The most commonly employed assessments for long-term morbidity in TBM are the modified Rankin Scale or Barthel Index, with recent meta-analyses reporting some physical disability in 32% of TBM survivors, using these tools ^1^. While the importance of severe disability is recognized, and often an endpoint in TBM clinical trials ^9^, these broad measures can miss the more subtle neurocognitive changes in TBM patients that can still impact overall wellbeing and economic output ^10^. Two Indian cohort studies used the Mini Mental Status Exam and found cognitive impairment in over half of survivors at six months and one year after TBM diagnosis ^11, 12^. Comprehensive neuropsychological testing using the Wechsler Adult Intelligence Scale in 17 TBM patients in Taiwan showed impairment in multiple domains including working memory and verbal comprehension ^6^. However, these studies in HIV-negative populations may not be representative of TB-HIV coinfection, as HIV, both independently and in conjunction with TB, contributes to neurocognitive impairment ^13, 14^; yet, TBM in HIV-infected persons is less inflammatory ^15^. Given recent findings of variability in reliability of cognitive assessments across different regional and cultural settings ^16^, it is essential that neurocognitive assessments are modified and standardized to local norms, as has been successfully applied in past studies of neurocognitive outcomes after cryptococcal meningitis ^17, 18^. Comprehensive neuropsychological testing has never been reported after TBM in an HIV-positive population or in sub-Saharan Africa. Furthermore, despite evidence of increased risk of mental illness in childhood survivors of TBM ^19^, the burden of depression in adult survivors of TBM is unknown.

Given the prevalence of disability in TBM survivors, further understanding of rehabilitation options is necessary. In 2017, the World Health Organization (WHO) identified rehabilitation as an increasing unmet need to address disability in low and middle income countries, and called for strengthening of these systems ^20^. In Uganda, availability of physiotherapy remains limited, and is often restricted to those with higher socioeconomic status and education ^21^. Neurorehabilitation has emerged as a specialized form of rehabilitation incorporating physiotherapy as well as occupational, speech, and psychiatric therapy, to target the potential for brain recovery in neurological diseases such as stroke and multiple sclerosis ^22^. Groups in India and West Africa have investigated telemedicine strategies for the rehabilitation of survivors of stroke, TBM, and other neurologic illnesses to overcome implementation barriers that exist in resource-limited settings ^23, 24^.

To better target neurorehabilitation resources, a clearer phenotype of the neurocognitive and functional impairment in TBM is necessary. In this nested prospective cohort study, we assessed detailed neurocognitive function, alongside depression and functional status, in Ugandan clinical trial participants who survived TB meningitis. To describe the cognitive deficits associated with TBM and their improvement over the first 6 months of recovery, tests were repeated at 8 and 24 weeks, and compared with a representative healthy control population.

## Methods

### Population and setting

Patients were enrolled in this prospective cohort from within the “ *High dose oral and intravenous rifampicin for improved survival from adult tuberculous meningitis*” (RIFT) study, a phase 2 open-label randomized trial (ISRCTN42218549) ^25^. Patients were enrolled in the parent trial between January 14 and December 17, 2019, at Kiruddu National Referral Hospital in Kampala, Uganda and Mbarara Regional Referral Hospital in Mbarara, Uganda, based on detection of TB in the cerebrospinal fluid (CSF) by Xpert MTB/RIF Ultra (Cepheid, Sunnyvale, CA) ^26^, or presentation compatible with TBM (CSF:plasma glucose ratio <50% or CSF glucose <65 mg/dL), coupled with TBM treatment planned. Exclusion criteria and study drug administration details are provided in the published trial protocol ^27^. We recorded baseline clinical data, CSF results, and demographics at initial presentation. Adjunctive corticosteroids were administered to all patients and antiretroviral therapy (ART)-naïve individuals initiated ART after completion of the intensive phase of TB treatment (week 8), in accordance with Ugandan guidelines (tenofovir/lamivudine/dolutegravir as first-line). HIV-positive participants also received cotrimoxazole prophylaxis.

We enrolled participants into this sub-study assessing neurocognitive and functional outcomes from the Kampala site eight weeks after their enrollment in the parent trial. We included those who survived the initial hospitalization and presented for their week eight post-randomization clinic follow-up visit. We excluded patients whose meningitis was later confirmed to be due to a non-TB etiology.

### Procedures

At week 8 and 24 visits, patients’ clinical status was recorded, as was their modified Rankin score and Karnofsky performance score, clinician-determined functional status measures. They were screened for depression using the patient health questionnaire (PHQ)-9 instrument, which ranges from 1 to 27 and has been validated in multiple countries in sub-Saharan Africa with a cutoff of 10 for moderate or severe depression ^28, 29^. We used a secondary cutoff of 15 to account for possible overlap in physical symptoms with TBM illness. As part of the visit, participants received a standardized battery of neurocognitive tests in either English or Luganda performed by a trained study nurse. The battery of tests evaluates ten neuropsychological and motor domains, and has been validated in sub-Saharan African populations and performed in Uganda on survivors of cryptococcal disease ^17, 30^. The WHO-University of California-Los Angeles Auditory Verbal Learning Test (WHO-UCLA AVLT) assesses verbal learning and memory ^31^, Digit Span Forward and Backward assesses attention and working memory ^32^, Semantic Verbal Fluency assesses language fluency ^33^, Timed Gait assesses gross motor function ^34^, Grooved Pegboard (average of both hands) assesses fine motor function ^35^, Finger Tapping (of the dominant hand) assesses motor speed ^36^, Symbol Digit Modality assesses processing speed and concentration ^37^, Color Trails 1 assesses processing speed and attention, and Color Trails 2 assesses executive function ^38^, ( Table 1).

**Table 1.** Neuropsychological test battery and neurocognitive domains evaluated.

Test	Test Description	Cognitive Domains
WHO-UCLA AVLT-Total*	Subjects are asked to recall a list of words. The test is similar to the Rey Auditory Verbal Learning test, however words have been selected to be recognizable to a variety of cultures	Verbal learning
WHO-UCLA AVLT-Delayed Recall*	Similar to WHO-UCLA AVLT, but subjects are asked to recall the same list of words in a delayed recall phase	Verbal memory
Digit Span Forward and Backward	Subjects are given a series of digits of increasing length and are asked to repeat them in forward or backward order	Attention, Working memory
Semantic Verbal Fluency	Subjects are given 60 seconds to produce as many words as possible within a specific category such as 'animals'	Language fluency (Verbal)
Symbol Digit Modality	Subjects are asked to match geometric figures to numbers as quickly as possible over 90 seconds using a visual reference.	Speed of information processing, Concentration
Color Trails 1	Subjects connect encircled numbers scattered on a page in sequence during a set amount of time. This test is similar to the Trail Making Test but has been formulated to minimize cultural bias by not using any letters or written instructions	Speed of information processing, Attention
Color Trails 2	Similar to The Color Trails 1 but each number is printed in two different colors, and subjects are asked to maintain the numerical sequence while alternating colors	Executive function
Timed Gait	The time for subjects to walk out and back 10 meters is recorded	Gross motor
Grooved Pegboard	Subjects are timed while placing pegs which each have a key along one side in holes in various orientations in a pegboard with either their dominant or non-dominant hand	Fine motor
Finger tapping	Subjects tap as rapidly as possible using the index finger on a specially adapted tapper for five 10-second trials	Motor speed

WHO-ULCA AVLT = World Health Organization-University of California-Los Angeles Auditory Verbal Learning test

### Statistical analyses

Raw scores on each test were standardized to duration of education (<7 years, 7 to 12 years, and >12 years) and age (greater or less than 30 years), matched to HIV-negative Ugandan controls (data collected as part of a prior neurocognitive study ^39, 40^) to create education- and age-adjusted Z-scores. To generate a global measure of neurocognitive function across all domains, a quantitative neurocognitive performance Z-score (QNPZ-8) was calculated as the mean of eight individual Z-scores: Symbol Digit, WHO-UCLA AVLT immediate and delayed recall, Verbal Fluency, Color trails 1 and 2, Finger Tapping, and Grooved Pegboard. We defined neurocognitive impairment as one standard deviation below the HIV-negative reference mean (corresponding to a Z-score of -1) and severe impairment as two standard deviations (Z-score < -2). Participants were permitted to skip tests if they started but were unable to complete it due to visual difficulties, fatigue, or physical limitations. Skipped tests were assigned Z-scores equal to the mean of the TBM cohort minus two standard deviations. All analyses were run on STATA version 15 (StataCorp, College Station, TX).

### Ethical considerations

Written informed consent was obtained from participants or their caregiver. The parent trial and this sub-study were approved by the Research Ethics Committees of LSHTM, UK, Mulago Hospital, Uganda National Council of Science and Technology, and Uganda National Drug Authority. An independent data safety committee reviewed accruing data from the parent trial.

## Results

### Cohort

**Figure 1.**
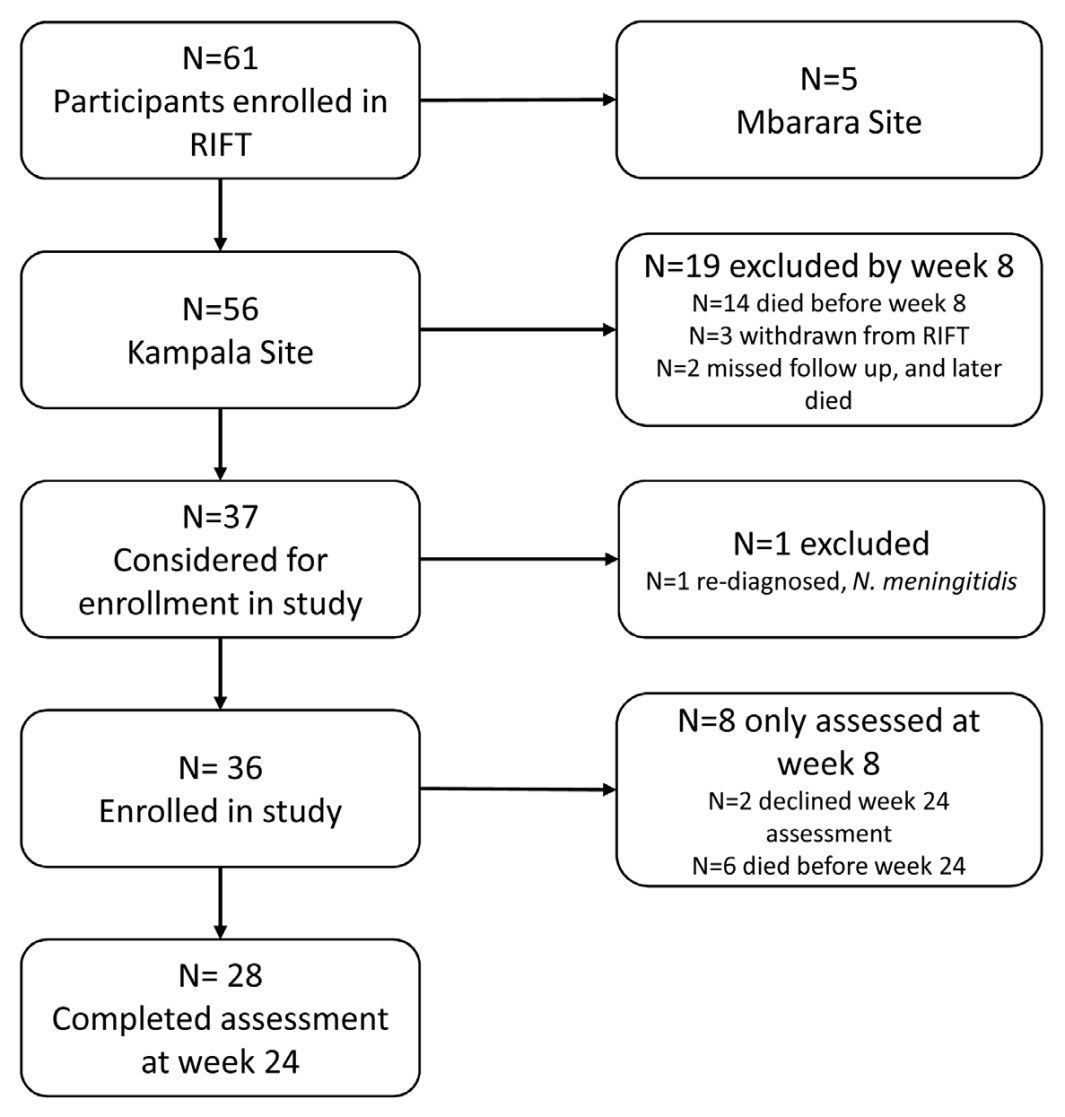
Enrollment in this nested sub-study from the parent randomized RIFT trial.

Of 56 patients enrolled in the parent trial at Kampala, 37 survived and remained at eight weeks follow-up to be considered for enrollment in this study ( Figure 1). The 19 not considered for enrollment either did not survive to week 8 (n=14), were withdrawn from the parent trial during the initial hospitalization (n=3), or were unable to present to their week 8 visit and later died (n=2). We enrolled 36 patients into the neurocognitive study after excluding one who had an alternate etiology of meningitis. Of the 36, 28 were reassessed at week 24 (n=6 died, n=2 declined assessment at week 24). 

Demographics and clinical data from the initial hospitalization are presented in Table 2. The cohort was relatively young (median age 35). Overall, 42% (15/36) had less than 7 years of education, 39% (n=14) had seven to 12 years of education, and 19% (n=8) had more than 12 years of education. Overall, 94% (34/36) were HIV-positive, and 44% (16/36) had microbiological-confirmed TBM. Due to low numbers in each experimental treatment group, and lack of statistically significant difference in the major neurocognitive and functional measures (QNPZ-8, modified Rankin Scale), neurocognitive data is described for the cohort as a whole rather than by randomized treatment group from the parent trial.

**Table 2.** Baseline results in persons with TBM.

Characteristics at Diagnosis	
Age, years	35 (29-37)
Women	18 (50%)
Education <7 years 7–12 years >12 years	15 (42%) 14 (39%) 7 (19%)
HIV-positive	34 (94%)
Receiving ART (of HIV-positive)	12 (35%)
CD4 count, cells/µL	111 (43-272)
CD4 count <200 cells/µL	19 (61%)
Microbiologically-confirmed TBM	16 (44%)
Trial Arm: Standard of Care High dose oral rifampin High dose IV rifampin	15 (42%) 10 (28%) 11 (31%)
TBM severity: MRC grade 1 2 3	4 (11%), 27 (75%), 5 (14%)
Glasgow Coma Scale score	14 (12-14)
CSF White Blood Cell Count, cells/µL	35 (<5-125)
CSF Protein, mg/dL	128 (94-177)
CSF Glucose, mg/dL	41 (21-68)
Serum Sodium, mEq/L	130 (126-136)

Values are medians with interquartile range (IQR) or N (%). ART: antiretroviral therapy, TBM: Tuberculous meningitis, MRC: medical research council grade, CSF: cerebrospinal fluid.

### Week 8 neurocognitive assessment

At eight weeks, 11 patients had at least moderate disability with a modified Rankin Scale score greater than or equal to 3 (median cohort score = 2, IQR 1-3), and 66% (19/29) of patients had Karnofsky scores <80, indicating inability to carry on normal activity ( Table 3).

**Table 3.** Week 8 and 24 neurocognitive and functional outcomes in persons with tuberculosis meningitis.

	Week 8	Week 24
modified Rankin Scale > 2	11 (31%)	3 (10%)
Karnofsky Functional Status Score < 80	19 (66%)	5 (19%)
PHQ-9 Depression Score ≥ 10	23 (85%)	18 (75%)
PHQ-9 Depression Score ≥ 15	19 (70%)	14 (58%)
QNPZ-8 Neurocognitive <-1 Z-score	31 (86%)	17 (61%)
QNPZ-8 Neurocognitive <-2 Z-score	19 (53%)	7 (25%)

Abbreviations: PHQ-9: patient health questionnaire 9

**Figure 2.**
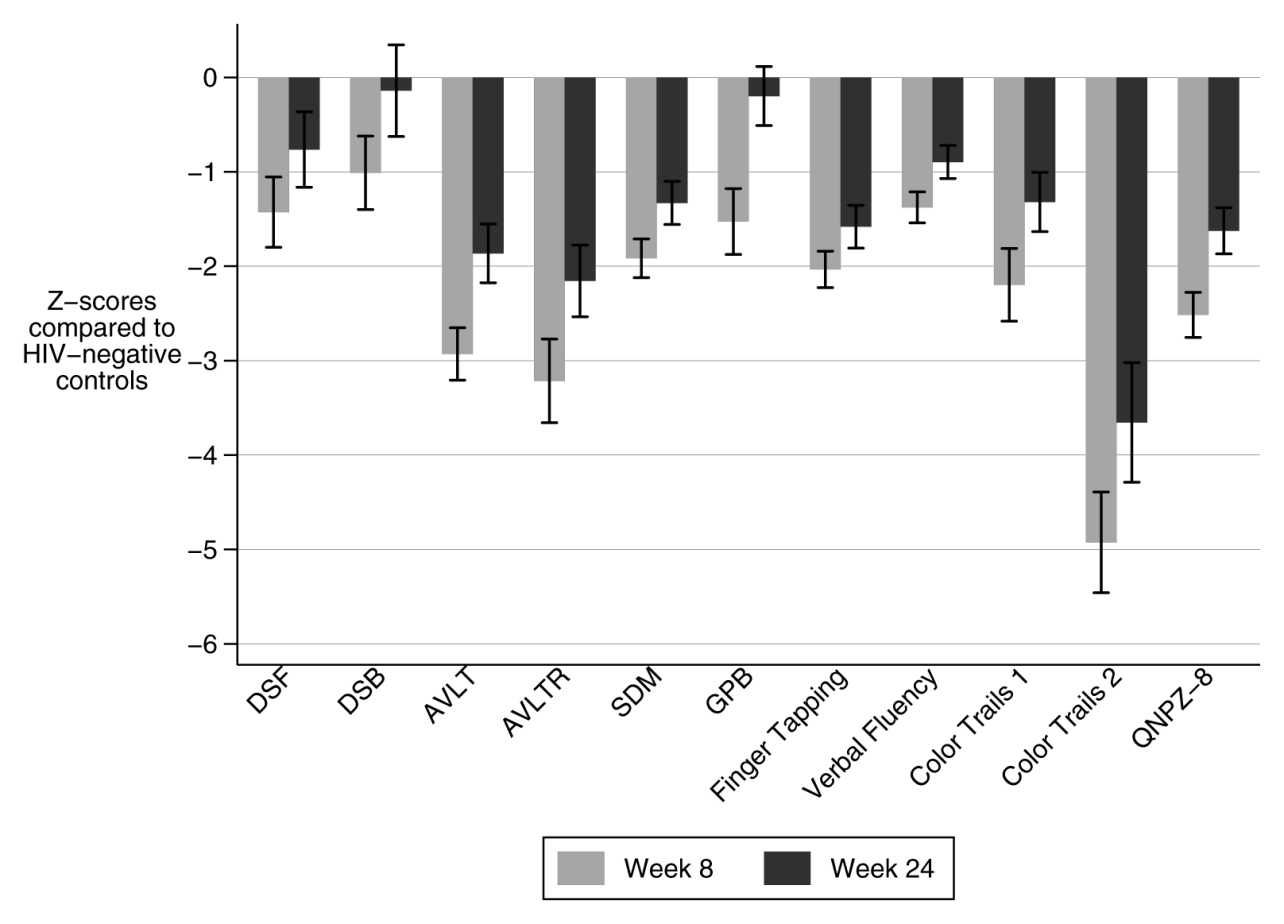
Impairment in neurocognitive domains at eight and 24 weeks in survivors of TBM. Mean cohort Z-scores in each neurocognitive assessment and the summary score (QNPZ-8) at both time points show improvement in most domains. A Z-score <-1 signifies impairment, and a Z-score <-2 signifies severe impairment. Error bars represent standard error. DSF: Digit Span Forward, DSB: Digit Span Backward, AVLT: WHO-UCLA Audio Verbal Learning Test Total, AVLTR: WHO-UCLA Audio Verbal Learning Test Recall, SDM: Symbol Digit Modality, GPB: grooved pegboard, QNPZ-8: Quantitative neurologic performance on eight modalities.

At week 8, 86% (31/36) of patients had impaired cognitive function (QNPZ-8 lower than -1), and 53% (19/36) had severe impairment (QNPZ-8 lower than -2). The mean QNPZ-8 score was -2.51 (standard deviation (±SD) ±1.43) representing 2.51 standard deviations (i.e. Z-score) below the population mean for HIV-negative Ugandans, adjusted for age and education. At eight weeks, impairment was nonspecific as all component assessments of the QNPZ-8 demonstrated cognitive impairment (Z-score < -1) on the cohort-level. Specific domains with severe impairment included executive function (color trails 2 assessment: -4.93, SD±3.20); verbal learning (AVLT-Total: - 2.93, SD±1.66); verbal memory (AVLT-Recall: -3.21, SD±2.66); and speed of information processing (color trails 1 assessment: -2.20, SD±2.31) ( Figure 2). While gross motor performance does not contribute to QNPZ-8, gross motor performance as assessed by timed gait was severely impaired, with a mean Z-score of -7.89 (SD±3.40).

### Week 24 neurocognitive assessment

At week 24, three patients had at least moderate disability (median modified Rankin = 0, IQR 0-1), and 19% (5/26) of patients had a Karnofsky score <80 ( Table 3).

**Figure 3.**
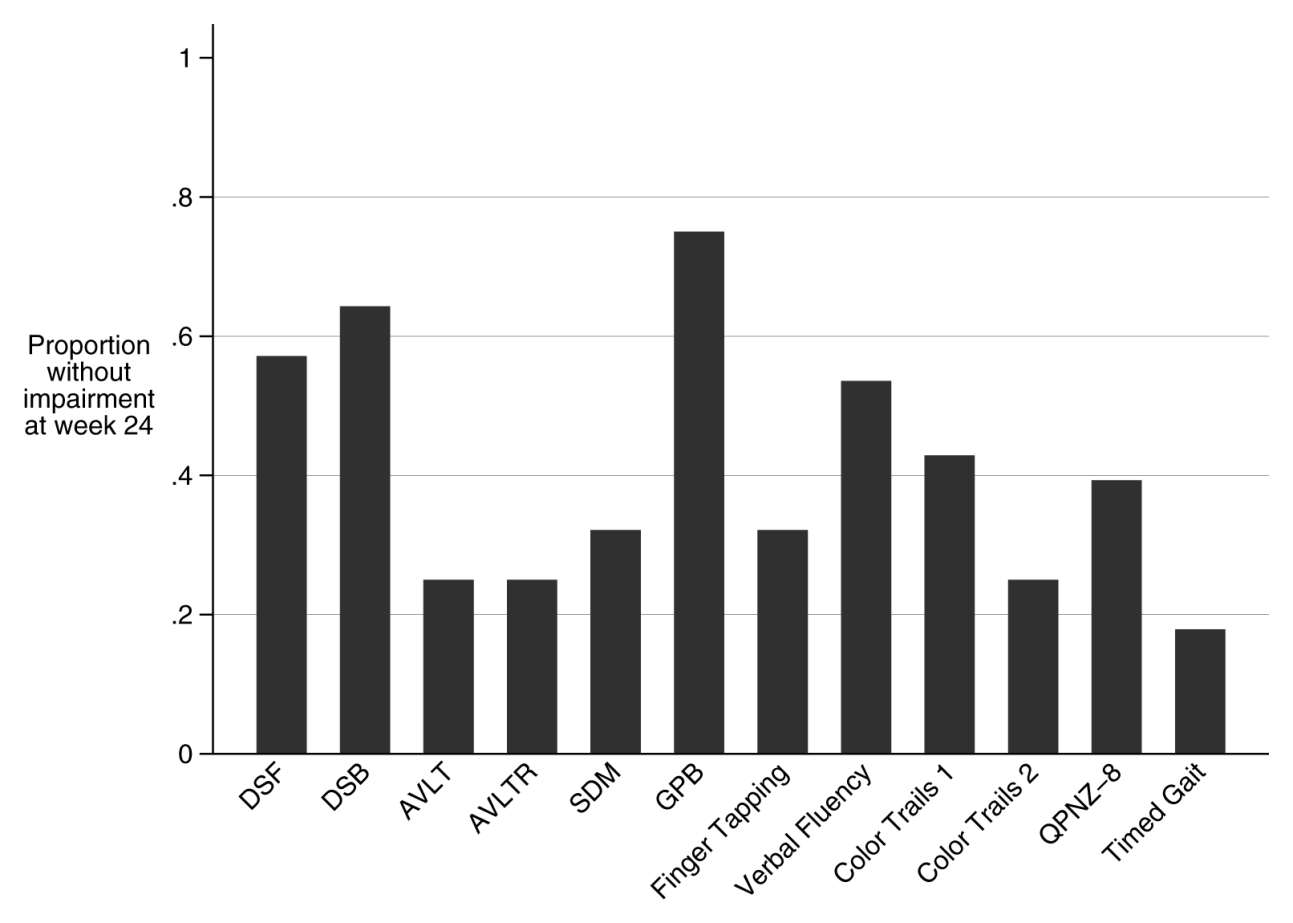
Proportions of the cohort that are no longer impaired in each assessment at week 24. Bars approaching 1 signify few participants with impairment in that domain. Majorities of the cohort have impairment in AVLT, AVLTR, SDM, Finger tapping, Color Trails 1, Color Trails 2, Timed Gait, and the summary score (QNPZ-8). Impairment on any given assessment is defined as a Z-score <-1. DSF: Digit Span Forward, DSB: Digit Span Backward, AVLT: WHO-UCLA Audio Verbal Learning Test Total, AVLTR: WHO-UCLA Audio Verbal Learning Test Recall, SDM: Symbol Digit Modality, GPB: grooved pegboard, QNPZ-8: Quantitative neurologic performance on 8 modalities.

At week 24, 61% (17/28) of patients had impaired cognitive function, and 25% (7/28) had severe impairment ( Figure 3). Mean QNPZ-8 at 24 weeks was -1.62 (SD ±1.29). Amongst the 28 patients tested at both time points, QNPZ-8 improved from a mean of -2.39 (SD ±1.52) to -1.62 (SD ±1.29). The most improved domains over these 16 weeks were fine motor (grooved pegboard, Z-score difference = 1.15) which improved to the mean of the HIV-negative Ugandan control group, and executive function (color trails 2 assessment, Z-score difference 1.32) which remained severely impaired ( Figure 2). Other domains which remained impaired were processing speed (color trails 1: -1.32 (SD ±1.66)) (symbol digit modality: -1.33 (SD ±1.21)), verbal learning (AVLT-total: -1.86 (SD ±1.65)), verbal memory (AVLT Recall: -2.16 (SD ±2.01)), and motor speed (finger tapping: -1.58 (SD ±1.20)). Color trails 1 and symbol digit modality also assess concentration and attention; however, these domains were judged unimpaired based on relatively normal results on tests of concentration and attention which do not test processing speed: digit span forward and backward (-0.76 SD ±2.1; -0.14 SD ±2.6 respectively). Timed gait remained severely impaired: mean Z-score was -5.11 (SD ±3.69).

Of note, seven patients at eight weeks, and two patients at 24 weeks were too ill to complete any test and therefore all scores including QNPZ-8 are imputed 2 standard deviations below the cohort mean. In a parallel analysis excluding these patients (see *Underlying data*), mean scores were slightly improved but relative differences between domains were similar in both populations.

### Depression screening

Moderate and severe depression, as defined by a PHQ-9 score ≥10 was present in a majority of the cohort (23/27 (85%) able to complete the questionnaire) at week 8. At week 24, rates of moderate and severe depression were somewhat lower (75%; 18/24), but still constituted a large majority of the cohort. Even with a higher cutoff (≥15), the majority screened positive for depression at both time points ( Table 3). Among the 21 who completed the questionnaire at both time points, moderate and severe depression was present in 17 (81%) at week 8, and 15 (71%) at week 24.

## Discussion

In this prospective study of 36 survivors of TBM in Uganda, we have reaffirmed the high degree of early functional disability present, demonstrated neurocognitive and functional improvement between two and six months, and described a phenotype of neurocognitive impairment predominantly in executive functioning, information processing speed, and verbal learning and memory. This phenotype is less apparent at eight weeks, when patients are often still recovering from their acute illness and are broadly impaired, but by 24 weeks becomes clear as some neurocognitive domains approach population norms while others remained impaired. Notably, at this time many patients were judged as clinically well and without significant disability (based on the modified Rankin Scale) by the study doctor, but there remained significant neurocognitive deficits that were identified on comprehensive neurocognitive testing. Longer follow-up is necessary to determine the durability of this impairment, and whether longer-term recovery is likely.

Many of the deficits identified were motor-related, including gross motor (timed gait), fine motor (grooved pegboard), and motor speed (finger tapping). Of the more explicitly cognitive domains, verbal learning and memory, processing speed, and executive function were especially affected. The deficits described mirror many of those found in a prior Taiwanese study (which did not test motor domains), where TBM survivors had significant deficits in processing speed (digit symbol), verbal comprehension (similarities), working memory (letter-number sequencing), and additionally, perceptual organization (block design, matrix reasoning) ^6^. While this suggests potential generalizability of TBM neurocognitive outcomes between HIV-positive and HIV-negative populations, further study is necessary.

Reflecting the epidemiology of TBM in Uganda ^41^, a majority of the cohort was HIV-positive and among those, a majority had a baseline CD4 T cell count <200 cells/µL, putting them at significant risk of HIV-associated dementia ^42^. Dissecting the neurocognitive impacts of HIV infection and TBM is inherently difficult, and current definitions of HIV-associated dementia exclude patients with central nervous system opportunistic infections like TBM ^43^. The typical profile of neurocognitive impairment in HIV-associated dementia includes deficits in verbal learning and memory, executive functioning, attention, and processing speed ^40, 44^. The deficits we described in TBM survivors in memory, executive functioning, and processing speed overlap this profile, although the additional deficits in gross motor domains, and relatively good performance in tests of attention not relying on speed, are notable. When the same battery of neurocognitive tests was administered to an HIV-positive cohort in Uganda ^40^, participants were impaired in verbal learning, gross motor, and executive function, but to a lesser degree than in this TBM cohort at 24 weeks (comparable Z-scores presented ^17^). This suggests neurocognitive impairment after TBM beyond what would be expected from HIV alone. ART improves symptoms of HIV-associated dementia ^30^, and 24 week testing on TBM survivors in our study (16 weeks after ART initiation) showed significant but far from complete improvement from baseline. Longer follow-up and evidence of immune recovery is necessary to better understand the contribution of HIV to the neurocognitive impairment after TBM.

We found a high prevalence of depression in survivors of TBM at both eight and 24 weeks. This is consistent with findings of high rates of depression in South African children with TBM ^19^. Interestingly, the rates of depression in this study are higher than in adult survivors of cryptococcal meningitis in Uganda (73% at one month in a 2010–2013 cohort, 62% in a 2015–2017 cohort) ^17, 45^. While there has been little study of the relationship between TBM and depression, the pathophysiology and treatment of TBM in our cohort involves HIV infection, inflammation, neurologic injury, and glucocorticoids, all of which are also associated with depression ^46– 50^. IL-6, known to play an important role in depression ^51– 53^, including inhibiting the serotonin pathway, is significantly associated with the severity of TBM ^54^. Cognitive impairment is a known symptom of depression ^55^, so some of the cognitive impairment seen in the cohort could be attributable to depression. As prior psychiatric illness was not assessed, we cannot determine whether premorbid depression may have also contributed to risk of advanced HIV and TBM. Given the association between depression and HIV-induced immunosuppression ^14^, it is notable that unlike the significant improvement in depression reported after ART initiation in survivors of cryptococcal meningitis ^17, 56^, high rates of depression persisted in our cohort at six months, well after ART was initiated. Immunologic differences in the response to cryptococcal meningitis and TBM ^54^, known to be important in the development and persistence of depression ^57, 58^, may partly explain this disparity. Differences between TBM and cryptococcal meningitis disease severity could further explain the difference in depressive symptoms, with TBM having higher rates of altered mental status while hospitalized ^45, 59– 61^, strokes ^7, 8, 62^, and persistent neurologic deficits. A comprehensive treatment of depression is essential to improve outcomes in TBM, and should be incorporated into follow-up and rehabilitation protocols.

The improvement in both motor and cognitive domains over six months is remarkable even without formal rehabilitation, but further recovery potential remains unknown. Given the predominance of motor impairment, physiotherapy could provide significant benefits, and deserves further study. More specialized rehabilitation practices might show benefit in the recovery from deficits in processing speed, executive function, and memory. Rehabilitation protocols designed for stroke survivors, which are the most available worldwide ^22, 24^, could be effective for TBM given that there is also a high prevalence of motor deficits, depression, and cognitive deficits (especially executive function and processing speed), although the exact phenotype of cognitive deficits differs depending on stroke location ^63, 64^. This population (median age 35 years) are in the most economically active period of life and thus rehabilitation may prove to be cost-effective. Further investment in local physiotherapy is essential in sub-Saharan Africa, but increasing experience with telemedicine provides an alternate method of care delivery ^23, 24^. Novel approaches, including brain-training video games, might be applicable for recovery from TBM as they have shown promise in improving working memory and processing speed in other populations ^65, 66^.

Strengths of this study include standardization of results to a locally representative cohort and detailed neurocognitive profiling at two time-points; limitations include the small cohort size and lack of follow up beyond 6 months. Larger studies will be necessary to investigate baseline risk factors for poor neurocognitive outcome.

Comprehensive neurocognitive testing of TBM survivors in sub-Saharan Africa is feasible. There is significant neurocognitive recovery between 2 and 6 months, but significant deficits remain in motor domains, as well as processing speed, verbal learning, and executive function. These findings highlight the need for neurorehabilitation and management of depression in TBM survivors.

## Data availability

### Underlying data

Repository name: Data Compass, https://doi.org/10.17037/DATA.00002372 ^67^

This project contains the following underlying data:

- Individual baseline results
- Individual clinical statuses at weeks 8 and 24
- Modified Rankin, Karnofsky performance,and PHQ-9 scores
- Individual raw scores for each test in the battery of neurocognitive tests
- Individual Z-scores on the neurocognitive tests

Data are available under the terms of the Data Sharing Agreement. Due to ethical considerations surrounding the sensitivity of the data in a vulnerable population, study consents limited the access to underlying data from this study. However, controlled access to the data posted in the above repository is permitted after signing of the agreement and IRB approval. Readers interested in the data can learn more by completing the application form on the Data Compass repository, or by contacting the LSHTM Research Data Management Service at researchdatamanagement@lshtm.ac.uk with the dataset DOI.
