# Supplementary material for: Neurocognitive outcomes of tuberculous meningitis in a primarily HIV-positive Ugandan cohort
Source: Wellcome Open Res. 2022 Mar 3;6:208. Originally published 2021 Aug 12. [Version 2] doi: 10.12688/wellcomeopenres.16967.2 (PMC9334841; doi:10.12688/wellcomeopenres.16967.2)
Supplement: Peer review history

# Peer review: Reviewer response for version 2

**DOI:** 10.21956/wellcomeopenres.19643.r51357

**Published:** 2022-07-28

**Authors:** Pablo F. Belaunzarán-Zamudio

**Affiliations:** 1 Division of AIDS, National Institutes of Allergy and Infectious Diseases, Mexico City, Mexico

This is an observational, cohort study nested within a phase II, open-label, clinical trial designed to test if high-dose rifampin administered intravenously or orally would improve blood and CSF rifampin exposure in people with tuberculosis meningitis (TBM) in a population consisting predominantly of people living with HIV (PLHIV) receiving care in Uganda. In this manuscript, Carson Quinn and colleagues report the results of longitudinal neurocognitive assessments performed in 36 participants of said trial that survived the initial event of TBM and had at least 8 weeks of follow-up. Only a fraction of these (n=28) completed the neurocognitive evaluations 24 weeks after enrollment. 

 This is a small, but relevant study providing evidence that people surviving TBM may have significant cognitive impairments 8 and 24 weeks after having initiated treatment for tuberculosis and around 4 months after ART initiation. While the participants improved over time in general, the phenotypes of impairment were remarkably different at each timepoint: from a non-specific phenotype with moderate to severe impairments in several areas (including executive function, verbal learning, verbal memory; and speed of information processing) at week 8, to a severe impairment of predominantly fine motor and executive functions at week 24. Remarkably, the authors also observed high prevalence of depression in this population, that did not seem to decrease significantly over time, which contrasted with significant improvements in disability.

 The article is well written, easy to follow, and the authors have provided, in my view, a coherent narrative of an important health problem with potential public health ramifications, on which not sufficient attention has been laid. The strength of the findings of the study is limited by the small sample size, relatively high attrition and short follow-up, and difficulties in measuring neurocognitive function, which the authors tried to address as best as possible and discussed in detail. Previous reviewers have pointed out several shortcomings, to which the authors responded adequately.

 I just want to add that non-acknowledged limitation of this study, is that this is a secondary analysis of a randomized clinical trial, with strict inclusion and exclusion criteria, that may have undiscussed implications for external validity, even locally. This is not a population study with a sample size or study population designed to answer the specific question that this analysis is trying to answer. Previous studies have shown important differences in sociodemographic characteristics and clinical outcomes between participants in clinical trials and people receiving care through routine clinical care. This does not undermine the relevance of this study, but needs to be acknowledged as a limitation.

 Also, while I agree with their conclusion that their findings highlight the need for neurorehabilitation and management of depression in TBM survivors; I would only add that larger studies with longer-term follow-up are needed to better characterize neurocognitive function outcomes in people recovering of tuberculous meningitis.

Is the work clearly and accurately presented and does it cite the current literature?

Yes

If applicable, is the statistical analysis and its interpretation appropriate?

Yes

Are all the source data underlying the results available to ensure full reproducibility?

Yes

Is the study design appropriate and is the work technically sound?

Partly

Are the conclusions drawn adequately supported by the results?

Yes

Are sufficient details of methods and analysis provided to allow replication by others?

Yes

Reviewer Expertise:

Epidemiology of HIV care, therapeutic clinical trials

I confirm that I have read this submission and believe that I have an appropriate level of expertise to confirm that it is of an acceptable scientific standard, however I have significant reservations, as outlined above.

# Peer review: Reviewer response for version 2

**DOI:** 10.21956/wellcomeopenres.19643.r48992

**Published:** 2022-03-04

**Authors:** Deanna Saylor

**Affiliations:** 1 Department of Neurology, School of Medicine, Johns Hopkins University, Baltimore, MD, USA; 2 University Teaching Hospital, Lusaka, Zambia; 3 MMED Neurology Program, School of Medicine, University of Zambia, Lusaka, Zambia

Dr. Quinn and team have adequately addressed all of my previous concerns, and I believe the revised manuscript has improved in clarity and in a more streamlined message.  I believe it represents a very important contribution to the TBM literature and congratulate the authors on this important work.

Is the work clearly and accurately presented and does it cite the current literature?

Yes

If applicable, is the statistical analysis and its interpretation appropriate?

Yes

Are all the source data underlying the results available to ensure full reproducibility?

Yes

Is the study design appropriate and is the work technically sound?

Partly

Are the conclusions drawn adequately supported by the results?

Yes

Are sufficient details of methods and analysis provided to allow replication by others?

Yes

Reviewer Expertise:

Neurocognitive impairment in HIV, neuro-infectious diseases, neuroepidemiology and clinical neurology in sub-Saharan Africa

I confirm that I have read this submission and believe that I have an appropriate level of expertise to confirm that it is of an acceptable scientific standard.

# Peer review: Reviewer response for version 1

**DOI:** 10.21956/wellcomeopenres.18727.r46454

**Published:** 2021-11-11

**Authors:** Reuben Robbins

**Affiliations:** 1 HIV Center for Clinical and Behavioral Studies, New York State Psychiatric Institute, New York City, NY, USA; 2 Columbia University, New York City, NY, USA

This is a well-written manuscript describing neurocognitive outcomes among a small sample of Ugandan adults with tuberculosis meningitis (TBM), most of whom were also HIV-infected. The study describes neurocognitive functioning at 8 and 24 weeks post enrollment in a prospective cohort, phase 2 label randomized trial for TBM. The authors found high rates of global and domain specific impairment at 8 weeks and slightly less rates of impairment at 24 weeks. This study makes an important contribution to our understanding of neurocognitive functioning in HIV-associated TBM.

 There are four concerns:

1. The title refers to HIV-associated TBM, yet two participants in the study do not have HIV. Why include them? Do they differ in any way from the HIV+ participants, whether demographically or neurocognitively?
2. The authors use the phrase "population mean" and "population norms" to describe the control sample's performance on the neuropsychological test battery. This is misleading, as a sample of 100 is not usually referred to as a population norm. Population based norms typically have sample sizes in the (many) thousands. These are control-based or convenience sample-based norms. They may or may not reflect the true Ugandan population performance on these tests. 
3. Since the sample of TBM patients receive the same battery of tests twice, could some of the improvements be explained by practice effects? Did the control sample, from which norms were derived, complete the battery of neuropsychological tests more than once? And if so, do the norms take practice effects into account. If not, this is a limitation and must be noted as such. 
4. There is little detail as to how the neuropsychological tests were adapted for Luganda. One of the source articles (citation #40) says, "All the tests had their instructions and content translated into Luganda." There is a growing body of literature describing some challenges of simple forward translations of neuropsychological tests. Without extensive cultural adaption that includes assessment of test acceptability and understandability that follow guidelines set forth by the International Test Commission and American Educational Research Association, it is not clear how "valid" the tests are for Uganda. That being said, because the battery has been used extensively there, the lack of information and procedures regarding adaptation can be noted as a limitation, and thus the tests may not accurately measure the cognitive domains they purport to and could bias performance downwards. 

Is the work clearly and accurately presented and does it cite the current literature?

Yes

If applicable, is the statistical analysis and its interpretation appropriate?

Yes

Are all the source data underlying the results available to ensure full reproducibility?

Yes

Is the study design appropriate and is the work technically sound?

Yes

Are the conclusions drawn adequately supported by the results?

Yes

Are sufficient details of methods and analysis provided to allow replication by others?

Yes

Reviewer Expertise:

Neuropsychology, HIV, test development and adaptation, international research

I confirm that I have read this submission and believe that I have an appropriate level of expertise to confirm that it is of an acceptable scientific standard, however I have significant reservations, as outlined above.

# Response

**Published:** 2022-02-25

**Authors:** Carson Quinn

Dear Dr. Robbins,

 We appreciate your thoughtful review and the recommendations you have made for improving the manuscript. We will respond to each of your comments below. A new revised version taking into account yours and Dr Saylor's comments has been submitted and should be available for review shortly. 

1. *The title refers to HIV-associated TBM, yet two participants in the study do not have HIV. Why include them? Do they differ in any way from the HIV+ participants, whether demographically or neurocognitively?* 
  - You bring up a very important point about our study population, and a manner in which the manuscript suffered from imprecise wording. In designing the study, we endeavored to enroll a population that was representative of the TB Meningitis burden in Uganda. Past studies of neurocognitive outcomes of TBM have enrolled patients from southern and southeast Asia, and we wanted to ensure patients in Sub-Saharan Africa were represented in this literature. This Ugandan TBM population is largely, but not entirely, an HIV-positive population. However, as you state, our title and other high-level descriptions of the study refer to the cohort as "HIV-positive" which is misleading. We have therefore changed our language to reflect that this is a "primarily HIV-positive cohort" and our goal was more so to reflect the population of TBM survivors in Uganda. Because there were only 2 HIV-negative participants it is challenging to definitively comment on group differences in outcomes or demographics. They were demographically similar to the cohort besides one being in the youngest quartile of age, and in outcomes they differed more from one another than from the cohort as a whole with one participant doing relatively well and the other poorly.
2. *The authors use the phrase "population mean" and "population norms" to describe the control sample's performance on the neuropsychological test battery. This is misleading, as a sample of 100 is not usually referred to as a population norm. Population based norms typically have sample sizes in the (many) thousands. These are control-based or convenience sample-based norms. They may or may not reflect the true Ugandan population performance on these tests. *
  - Thank you for pointing out this imprecise language on our part. We have adjusted the wording to refer to the “control group mean” rather than population mean and added the limited size of our control group as a limitation.
3. *Since the sample of TBM patients receive the same battery of tests twice, could some of the improvements be explained by practice effects? Did the control sample, from which norms were derived, complete the battery of neuropsychological tests more than once? And if so, do the norms take practice effects into account. If not, this is a limitation and must be noted as such. *
  - *​​​​​​​*This is another excellent point. Practice effects certainly could have played a role, as the control sample to which we have normed the results did not have participants take the battery more than once. However, we hoped to lessen these effects by designing the study with a relatively long time between test administrations (4 months). However, as it could have still played a role, we have added this as a limitation to the study.  
4. *There is little detail as to how the neuropsychological tests were adapted for Luganda. One of the source articles (citation #40) says, "All the tests had their instructions and content translated into Luganda." There is a growing body of literature describing some challenges of simple forward translations of neuropsychological tests. Without extensive cultural adaption that includes assessment of test acceptability and understandability that follow guidelines set forth by the International Test Commission and American Educational Research Association, it is not clear how "valid" the tests are for Uganda. That being said, because the battery has been used extensively there, the lack of information and procedures regarding adaptation can be noted as a limitation, and thus the tests may not accurately measure the cognitive domains they purport to and could bias performance downwards. *
  - Thank you for bringing up this important point that is essential to consider when undertaking neurocognitive testing on non-English speaking participants from cultural contexts unlike those of the participants the test was initially designed for. We were unable to obtain information on exactly how the tests were adapted for Luganda initially by our collaborators. However, this battery of tests, and the International HIV Dementia Scale one which they are based, have been validated in past studies with Luganda-speaking Ugandan participants. Other studies have also assessed differences in test results between English and Luganda-speaking participants and found them to be minimal. For these reasons, this battery is commonly used in Uganda, and we are reassured that language differences are unlikely to be significantly biasing our results. This is now better-explained in the manuscript with appropriate citations, and added as a limitation.

*Addendum to Response to Reviewer 1*

 After further consideration of the matter of treatment arm differences in outcomes, we have determined that inclusion of further data on neurocognitive outcomes between the different treatment arms would be counterproductive, as the intention of the study was to describe neurocognitive outcomes in TB meningitis not to test the impact of the trial treatment. With the low numbers in each group, no meaningful conclusions could be expected to be drawn. Differences in functional status and other clinical outcomes between the trial arms are included in the manuscript for the parent trial. This is now expounded upon further in the manuscript text, and noted as a limitation inherent in our small cohort.

# Peer review: Reviewer response for version 1

**DOI:** 10.21956/wellcomeopenres.18727.r45499

**Published:** 2021-09-20

**Authors:** Deanna Saylor

**Affiliations:** 1 Department of Neurology, School of Medicine, Johns Hopkins University, Baltimore, MD, USA; 2 University Teaching Hospital, Lusaka, Zambia; 3 MMED Neurology Program, School of Medicine, University of Zambia, Lusaka, Zambia

Quinn, et al highlight the knowledge gap regarding neurocognitive and psychiatric outcomes in people with HIV (PWH) who survive TB meningitis (TBM) overall and in sub-Saharan Africa in particular and set out to determine these outcomes in a group of PWH TBM survivors in Uganda. They found nearly uniform neurocognitive impairment at 8 weeks, which improved but remained substantial at 24 weeks. In particular, verbal learning and memory, processing speed, and executive function were impaired as well as multiple domains of motor function. The authors also found high rates very high rates of depression at 8 weeks, and this did not change significantly at 24 weeks. Overall, this is an important study trying to address an important knowledge gap in the literature. However, there are important methodological and analytical decisions that were made which need to be further explained and/or reconsidered in order to further strengthen the paper. Finally, I believe a more nuanced consideration of the limitations of the study is also needed. Specifically:

1. The authors state that the goal of the study was to understand post-TBM depression and cognitive outcomes in PWH living in sub-Saharan Africa. As such, it is surprising to me that the two HIV-uninfected participants were included in the study. These patients are likely to be quite different than those with HIV co-infection and increase the heterogeneity of the population and data obtained. Given that they represent such a small proportion of the study cohort, I would strongly consider excluding them from the analysis or, at minimum, provide a strong justification for why they should remain.
2. Please expand more on how the possibility that the treatment received in the trial may have contributed to neurocognitive and/or depression outcomes was assessed. It may also be prudent to list the possibility that this was not able to be completely accounted for in the analysis due to small sample sizes in each treatment group as a possible limitation.
3. Please provide greater detail about the demographics of HIV-uninfected controls used for neurocognitive norms. Was the language of administration similar? Were education levels distributed somewhat similarly? This is important in understanding the validity of the normative data used for the study cohort and may also be a limitation of the study.
4. Please justify the decision to include patients who were too sick to complete the analyses in the neurocognitive outcomes but not the depression outcomes. Do the authors really think including these patients is representative of the typical neurocognitive deficits after TBM? At a minimum, it would be great to present more detail about how results differed when excluding them.
5. It appears there is a significant number of participants with missing data on functional outcome measures at 8 weeks. Can this be explained?

Is the work clearly and accurately presented and does it cite the current literature?

Yes

If applicable, is the statistical analysis and its interpretation appropriate?

Yes

Are all the source data underlying the results available to ensure full reproducibility?

Yes

Is the study design appropriate and is the work technically sound?

Partly

Are the conclusions drawn adequately supported by the results?

Yes

Are sufficient details of methods and analysis provided to allow replication by others?

Yes

Reviewer Expertise:

Neurocognitive impairment in HIV, neuro-infectious diseases, neuroepidemiology and clinical neurology in sub-Saharan Africa

I confirm that I have read this submission and believe that I have an appropriate level of expertise to confirm that it is of an acceptable scientific standard, however I have significant reservations, as outlined above.

# Response

**Published:** 2021-10-15

**Authors:** Carson Quinn

Dear Dr. Saylor,

 We appreciate your thoughtful review and the recommendations you have made for improving the manuscript. We will respond to each of your comments below:

1. *The authors state that the goal of the study was to understand post-TBM depression and cognitive outcomes in PWH living in sub-Saharan Africa. As such, it is surprising to me that the two HIV-uninfected participants were included in the study. These patients are likely to be quite different than those with HIV co-infection and increase the heterogeneity of the population and data obtained. Given that they represent such a small proportion of the study cohort, I would strongly consider excluding them from the analysis or, at minimum, provide a strong justification for why they should remain.* You bring up a very important point about our study population, and a manner in which the manuscript suffered from imprecise wording. In designing the study, we endeavored to enroll a population that was representative of the TB Meningitis burden in Uganda. Past studies of neurocognitive outcomes of TBM have enrolled patients from southern and southeast Asia, and we wanted to ensure patients in Sub-Saharan Africa were represented in this literature. This Ugandan TBM population is largely, but not entirely, an HIV-positive population. However, as you state, our title and other high-level descriptions of the study refer to the cohort as "HIV-positive" which is misleading. We will therefore change our language to reflect that this is a "primarily HIV-positive cohort" and our goal was moreso to reflect the population of TBM survivors in Uganda.
2. *Please expand more on how the possibility that the treatment received in the trial may have contributed to neurocognitive and/or depression outcomes was assessed. It may also be prudent to list the possibility that this was not able to be completely accounted for in the analysis due to small sample sizes in each treatment group as a possible limitation. *You are correct in wondering how the trial treatment could have impacted neurocognitive outcomes in survivors, and it is a question that we intend to study further in the future. This study was not powered to answer this question, and the numbers in each of the treatment groups that survived to neurocognitive assessment follow-up is too low to meaningfully comment on the differential impact of the trial treatments. However, in these small groups, there did not appear to be any significant difference in the summary neurocognitive score between the treatment groups. We will ensure that this data is provided in the revised manuscript (it is currently present in the extended data that is available), and comment on this accordingly as a limitation of the manuscript.
3. *Please provide greater detail about the demographics of HIV-uninfected controls used for neurocognitive norms. Was the language of administration similar? Were education levels distributed somewhat similarly? This is important in understanding the validity of the normative data used for the study cohort and may also be a limitation of the study.* The HIV-negative control participants are cohorted by age and education in the same groups as our study population to allow for norming of the study data. The tests were also performed in Luganda and English according to participant preference for both the controls and study population. We will provide more complete demographics beyond this in the revised manuscript.
4. *Please justify the decision to include patients who were too sick to complete the analyses in the neurocognitive outcomes but not the depression outcomes. Do the authors really think including these patients is representative of the typical neurocognitive deficits after TBM? At a minimum, it would be great to present more detail about how results differed when excluding them.* The decision to include participants too sick to complete the neurocognitive assessment was one of the major challenges in designing this study; while both options (to include or not to include) had drawbacks, I will provide our rationale here and comment further in the revised manuscript. Our intended population was survivors of TB meningitis -- many of these survivors are unfortunately very functionally impaired and remain with a significant burden of chronic symptoms from their TBM. To exclude these patients would be to represent TBM as a less morbid disease than it in fact is. It is true that the imputed values for the neurocognitive assessments in these patients aren't particularly meaningful on an individual level, and do not further our goal of demonstrating a typical phenotype of impairment in TBM. However, they do serve to accurately represent the degree of neurocognitive impairment frequently present in survivors of TBM. We did perform a sensitivity analysis showing that inclusion of these patients did not importantly impact the neurocognitive domains affected. We will be sure to add this data to the revised manuscript and explain this limitation further. Furthermore, we hope for this study to contribute to a standardized format allowing cross-comparison for future studies of neurocognitive impairment in TBM, which may have goals of investigating risk factors for impairment -- in which case inclusion of these more impaired patients is essential. 
5. *It* *appears there is a significant number of participants with missing data on functional outcome measures at 8 weeks. Can this be explained?* Thank you for noting this. This Karnofsky functional score were missing in patients deemed too impaired to complete neurocognitive assessment; however, as those scores are in fact available, they will be added to the revised manuscript. 

Thank you again for your many constructive comments. We intend to submit a revised manuscript after receiving a second review so as to respond to comments from both reviewers. I am hopeful that the revised manuscript appropriately addresses your concerns. 

 Sincerely,

 Carson Quinn on behalf of the authors
